# Targeting Heterogeneous Findings in Neuronal Oscillations in Tinnitus: Analyzing MEG Novices and Mental Health Comorbidities

**DOI:** 10.3389/fpsyg.2018.00235

**Published:** 2018-03-02

**Authors:** Pia Lau, Andreas Wollbrink, Robert Wunderlich, Alva Engell, Alwina Löhe, Markus Junghöfer, Christo Pantev

**Affiliations:** Institute for Biomagnetism and Biosignalanalysis, University Hospital of Münster, Münster, Germany

**Keywords:** tinnitus, neuronal oscillations, spectral analysis, magnetencephalography, auditory cortex

## Abstract

Tinnitus is a prevalent phenomenon and bothersome for people affected by it. Its occurrence and maintenance have a clear neuroscientific tie and one aspect are differences in the neuronal oscillatory pattern, especially in auditory cortical areas. As studies in this field come to different results, the aim of this study was to analyze a large number of participants to achieve more stable results. Furthermore, we expanded our analysis to two variables of potential influence, namely being a novice to neuroscientific measurements and the exclusion of psychological comorbidities. Oscillatory brain activity of 88 subjects (46 with a chronic tinnitus percept, 42 without) measured in resting state by MEG was investigated. In the analysis based on the whole group, in sensor space increased activity in the delta frequency band was found in tinnitus patients. Analyzing the subgroup of novices, a significant difference in the theta band emerged additionally to the delta band difference (sensor space). Localizing the origin of the activity, we found a difference in theta and gamma band for the auditory regions for the whole group and the same significant difference in the subgroup of novices. However, no differences in oscillatory activity were observed between tinnitus and control groups once subjects with mental health comorbidity were excluded. Against the background of previous studies, the study at hand underlines the fragility of the results in the field of neuronal cortical oscillations in tinnitus. It supports the body of research arguing for low frequency oscillations and gamma band activity as markers associated with tinnitus.

## Introduction

Tinnitus is a phantom auditory percept, which exists unrelated to any external source. It is – at least temporarily – familiar to up to 30% of the population and affects up to 14% as a chronic condition (Axelsson and Ringdahl, 1989; Sindhusake et al., 2003). Next to its perception as being annoying, between 1 and 3% of all people report a meaningful decrease in quality of life through the tinnitus, which can be expressed for example in sleeping disorders or depressed mood (Dobie, 2003). Hearing loss is strongly associated with tinnitus and triggering underlying maladaptive subcortical and cortical processes such as hypersynchronicity, hyperactivity, and burst firing (Shore et al., 2016). Markers of ongoing cortical activity are neuronal oscillations which represent the “rhythmic fluctuations in the excitability of neurons or populations of neurons” (Cohen, 2014).

Several studies in the past focused on neuronal oscillations in tinnitus (see Zobay et al., 2015) and tried to establish theories based on their results. Weisz et al. (2005) found an increase in delta and a decrease in alpha frequency band over temporal regions, nicely fitting into the established framework of alpha as a mechanism of an inhibitory balancing rhythm (Jensen and Mazaheri, 2010). A lack of alpha power – especially in the temporal regions where the auditory cortex is located – might thus represent a lack of inhibition in these areas. This might correspond to the above mentioned hyperactivity and increased spontaneous firing rate of the auditory cortex. Additional support for the idea of alpha oscillations as a key mechanism for tinnitus comes from research on comparable phenomena such as auditory hallucinations: Müller et al. (2014) for instance presented popular melodies to their subjects. In short gaps in the music, which were filled with pink noise, subjects who reported that they had the impression the melody would continue, showed decreased levels of alpha activity compared to those who did not report that the melody continued for them. Leske et al. (2014) demonstrated that the strength of the illusionary percept of the Zwicker tone negatively correlated with alpha frequency band power. Therefore, both studies show some form of reduced alpha activity in auditory areas if an auditory illusion is present. Recent neurofeedback studies further support this framework (Hartmann et al., 2014): if tinnitus patients were able to alternate, e.g., increase the power of alpha oscillations in their temporal areas of their brain, their tinnitus percept was altered as well, as it got more quiet. Despite its appealing and intuitive character of the findings in the alpha power band, results are not conclusive, as there are studies which do not report any anomalies in the alpha spectrum of tinnitus patients (Adjamian et al., 2012).

Two other prominent models explaining the role of neuronal oscillations in tinnitus make assumptions about theta/delta and gamma oscillations. One model thereby focuses on thalamocortical dysrhythmia (TCD, Llinas et al., 1999; Adjamian et al., 2012; De Ridder et al., 2015). A disrupted communication between the thalamus and cortical areas, that results in symptomatology, is the central idea of this model. This pathological communication is thought to be expressed in an increase in the theta and gamma activity (Llinas et al., 1999; Adjamian et al., 2012; De Ridder et al., 2015). The other model focuses on predictive coding, a current trend in neuroscience. This approach assumes that the brain is constantly producing predictions of the input and if those predictions are violated a prediction error is encoded. Sedley et al. (2016) state that spontaneous activity in the sub-cortical auditory pathway, the so-called tinnitus precursor, is existent but usually neglected in favor of the concept “silence.” Tinnitus emerges, if the prediction and the precursor converge. This means, the concept or prediction of silence is abandoned. This occurs, if the precursor gains precision and lastly replaces the prediction “silence.” Another option is, that the precursor codes a more intense tinnitus than the prediction would forecast. So in both cases, no-tinnitus and (less intense) tinnitus, the model would anticipate a prediction error, as there is a discrepancy between the prediction (silence or less intense tinnitus) and the precursor. This prediction error reflects a suppression of the tinnitus. Following this hypothesis, in case of tinnitus the prediction error would be reduced as prediction and precursor match. Their model also covers the specific prediction of neuronal oscillations: low frequencies as theta or delta and gamma oscillations, representing the incident of prediction errors, are expected. There is also some support for these suppositions in the research body of tinnitus, yet the results are still quite heterogeneous (Zobay et al., 2015). Taken together, results of previous studies offered heterogeneous results, which can be divided in evidence favoring the model of an importance of alpha oscillations, as well as models based on TCD or predictive coding, which underline the importance of low frequency oscillations.

Possible reasons for this heterogeneity in the different studies are hard to trace. They could represent systematic changes between the compared groups, yet influences through many other factors are likely, which may smear or veil the true effects. An overview of additional technical factors and aspects regarding data handling and data analysis of oscillatory brain activity, which could affect the results, are covered in a paper by Gross et al. (2013). Besides these technical and analyses-specific aspects pointed out in that paper, also biological factors [e.g., arousal level, hearing loss (Adjamian et al., 2012), the menstrual cycle (Brötzner et al., 2014)] as well as psychological variables (instruction, state of mind, mental health status) may affect neuronal oscillations. Additionally, sample sizes are varying between studies and are often considered to be too small (cf. Adjamian, 2014). In general the question was raised, if healthy subjects and patients have the same experience with the performed measurements. Experienced participants (e.g., persons who participated in multiple experiments) might show less arousal, less vigilance, than unexperienced participants leading to differential oscillatory resting state activity such as reduced alpha. A need to quantify those possible differences was acclaimed (Diaz et al., 2013). This question is of special importance for the research on neural oscillation in tinnitus, as tinnitus patients are often novices to the MEG and the healthy controls often function as control subjects on a regular basis for different studies and therefore they are no novices to the MEG measurement any more.

Of course, those confounding variables are always present in neuroscientific research. It is typically assumed that those variables are equally distributed between the groups and therefore their veiling effect is canceled out. However, in resting state measurements they may be more influential, as the measured signal is more subtle than for example evoked brain activity. Past research has shown, that there is powerful and meaningful output arising from this mode of measurement (resting state) in basic as well as clinical research (e.g., see Başar and Güntekin, 2008). Electrophysiological resting state measurements in tinnitus might be even more interesting to look at as these measurements are with high ecological validity as they are typically recorded in rather quiet surroundings without external activity/stimulation (e.g., no external source, permanently present).

Another important factor, which might add to the heterogeneity of the results in the research on tinnitus, are psychological comorbidities. Those are high in tinnitus patients (Zirke et al., 2010) and some comorbidities are known to have oscillatory correlates (e.g., for Depression: Jiang et al., 2016, for bipolar disorder: Degabriele and Lagopoulos, 2009). In tinnitus research is a focus on identifying underlying neural circuits of tinnitus distress and depression. For the former correlates in high alpha and beta band have been found, especially in right frontal areas, and for the latter in some studies alpha band correlations, here more left frontal lateralized; both constructs have a presumed neuronal overlap in parahippocampal areas (Vanneste et al., 2010; Joos et al., 2012; Meyer et al., 2017). Thus, another aim of the study at hand was to evaluate the impact of comorbid psychological disorders or rather the effect left, if psychological comorbidities in the sample are ruled out. In clinical research, conducting a DSM (Diagnostic and statistical Manual of Mental Disorders) based semi-structured interview covering psychological disorders (SKID, German version, Wittchen et al., 1997) is considered to be the gold standard for detecting and assessing mental health. Therefore, we screened a subgroup of tinnitus patients as well as healthy controls for psychological comorbidities with this instrument in order to detect any mental health disorders.

Taken the current body of research, we tried to shed more light onto the neuronal oscillations in tinnitus based on (a) a sufficiently large sample size (b) by approximation of potential confounding variables of being a novice in the measuring procedure and (c) by ruling out any mental health comorbidity.

## Materials and Methods

### Subjects

Fifty-nine participants having chronic tinnitus were recruited and measured. Due to artifacts (e.g., eye blinks, muscle artifacts), 13 dropped out, leaving 46 participants (mean age ± standard deviation: 41.59 ± 9.92 years; 46% female) in the tinnitus group for data analysis. Fifty-seven healthy controls were recruited, in 15 cases the data did not meet our requirements for analysis (i.e., 90 artifact-free trials) so we conducted our analysis with 42 healthy control participants without tinnitus (39.52 ± 12.05 years; 62% female). There was no significant group difference regarding age and gender (*p* > 0.05). The study protocol was approved by the ethics committee of the Department of Psychology of the University of Münster and was conducted according to the Declaration of Helsinki. Each participant signed informed consent prior to the investigation. Recruitment was conducted through advertisements in local newspapers, flyers in public places, doctor’s offices (ENT) and our homepage.

#### Subgroup of Novices

Twenty-nine subjects with a chronic tinnitus perception (mean age ± standard deviation: 39.00 ± 10.69 years; 48% female) and 26 healthy control participants without any tinnitus perception (42.65 ± 11.95 years; 62% female) were analyzed as a subgroup. All subjects participated for the first time in a MEG measurement. There was no significant group difference regarding age and gender (*p* > 0.05) between these subgroups.

#### Subgroup of Psychological Comorbidity Free Subjects

**FIGURE 1:**
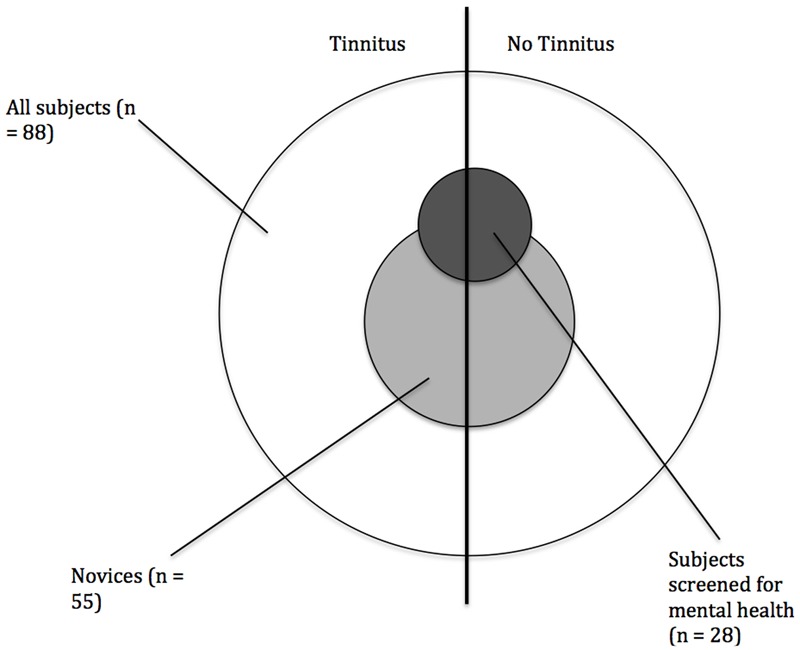
Scheme of the organization of the subjects.

Out of the whole sample 40 subjects (20 per group) were screened for psychological disorders with the SKID. Two participants of the tinnitus group fulfilled the criteria of a mental health disorder (Major depressive Episode, Alcohol Abuse) and were therefore excluded from analysis. The other drop-outs (*n* = 10) were eliminated as their artifact free number of epochs was not sufficient. So 14 subjects with tinnitus (44.36 ± 13.45 years; 50% female) and 14 subjects without tinnitus (42.86 ± 10.26 years; 43% female) having no psychological disorder were analyzed. There was no significant group difference regarding age and gender (*p* > 0.05). For a graphical overview of the different groups analyzed in this paper see **Figure 1**.

### Procedures

Five minutes resting state MEG recordings were collected by means of a 275 channel whole-head MEG system (OMEGA 2005 WC, CTF Systems, Inc., Port Coquitlam, BC, Canada) with first order axial gradiometers placed in a magnetically shielded and acoustically quiet room. The participants sat upright in a comfortable position; head position was supported through cotton pad stabilization in the dewar and constantly monitored. During data acquisition participants were instructed to look at a black screen with their eyes open [52 cm × 40 cm (WxH)] at a distance of 90 cm and ‘to relax doing nothing.’ A continuous data stream was acquired and digitally sampled at a rate of 600 Hz. The 5 min recordings of resting state were always performed prior to further MEG acquisitions used for other studies (Stein et al., 2014; Wunderlich et al., 2015; Domschke et al., 2016; Zwanzger et al., 2016).

### Preprocessing and Data Analysis

#### Sensor Space

The recorded data were analyzed using the FieldTrip toolbox (Oostenveld et al., 2011) under Matlab (The MathWorks, Natick, MA, United States, Version R2013b). An independent component analysis by means of “runica algorithm” (Makeig et al., 1997) was performed in order to identify the strongest components corresponding to ocular, cardiac, and muscle artifacts. Based on their statistical performance, the individual independent components for each subject were rejected from the data in an automated procedure (Dammers et al., 2008). The continuous data stream was then segmented into 150 epochs of 2 s each. A 50 Hz notch filter (plus its harmonics) was applied to eliminate disturbances by the electric power supply. In order to analyze relevant brain waves, an offline 4^th^ order Butterworth low-pass filter (cut-off frequency of 45 Hz) was applied to the data that were DC offset corrected based on the whole epoch length. Additionally, trials containing channels with a signal range larger than 2.5 pT were regarded as artifact-contaminated and excluded. Ninety of the remaining trials were randomly chosen for further analysis. Participants with a residual trial number below 90 were excluded. To account for intra-individual head positions during the MEG measurements, the data was projected onto a common sensor space (Knösche et al., 2002). Axial gradiometer recorded data were further transformed to planar gradiometers by taking the norm of the first spatial derivatives in polar and azimuthal directions for each sensor (Knösche and Bastiaansen, 2002). This was done for a better interpretation of the location of the activation picked up by the coils.

In the following, a spectral analysis was performed by means of a fast Fourier transformation (FFT) between DC and 30 Hz after applying a Hanning window to each epoch. Then, for each subject the power spectral density averaged across epochs was normalized by the individual overall spectral power (power spectral density averaged across all channels and frequencies).

#### Source Space

As most models have a prediction of neuronal activity in the auditory cortex (Zobay et al., 2015), we restricted the source space analysis to this area. To analyze the neuronal activity in the estimation of the auditory areas, the Brain Electrical Source Analysis software (BESA Research 6.0, Megis Software) was used. The recorded data was imported into BESA. In order to circumvent referring to data being processed by FieldTrip routines for the sensor space analysis, the data preprocessing has been repeated for the source space analysis using almost the same steps in BESA. Thereby we followed the FieldTrip approach described above as closely as possible. To eliminate artifact activity as eye blinks, movements or cardiac activity from the brain activity, an ICA was calculated as implemented in BESA (Lee et al., 1999). Independent component analysis using an extended infomax algorithm for mixed subgaussian and supergaussian sources was used (Lee and Lewicki, 2002) and identified artifact contaminated components were subtracted from the raw data. Data was again cut into epochs of 2 s and 1 Hz high pass and 180 Hz low pass filters were applied to the data. Trials with an amplitude range over 2.5 pT at any MEG sensor were regarded as contaminated and rejected.

Based on a source montage template for auditory brain regions (three temporal sources in the left and three symmetric sources in the right hemisphere; see also **Supplementary Figure S1**), we reconstructed the source strength waveforms (cf. Scherg et al., 2002) using BESA. This auditory source montage is based on literature on auditory activity (an overview of the exact procedure is provided in Scherg et al., 2002). Afterward a FFT frequency analysis was calculated for each source comprising the source montage. The obtained normalized power spectrum was exported to SPSS (IBM SPSS Statistics for Macintosh, Version 24.0) for further statistical analysis.

### Statistical Analysis

#### Sensor Space

To check for group differences (tinnitus vs. no tinnitus) in the *a priori* defined frequency bands {delta [1–4 Hz], theta [5–7 Hz], alpha [8–12 Hz] (low alpha [8–10 Hz] and high alpha [10–12 Hz]), beta [13–30 Hz], gamma [31–45 Hz]}, non-parametric statistical tests were calculated. Cluster-based statistics were calculated to determine significance probabilities based on a permutation distribution (Monte Carlo). For each frequency band 1000 permutations were drawn. The Monte Carlo *p*-values were set to *p* < 0.025 on sensor and *p* < 0.05 on cluster level and the used statistic for the permuting were an independent *t*- tests.

These analyses were done for the whole group comparison (1), the subgroups of novices (2) and psychological comorbidity-free subjects (3) (all: tinnitus vs. no tinnitus).

#### Source Space

The spectral components of the temporal source montage extracted from the BESA analysis were averaged over the six individual sources in the auditory area for each frequency band and normalized (over all auditory sources and frequencies). Using a one-way ANOVA in SPSS we compared the frequency bands. As we tested for seven frequency bands, all *p*-values were Bonferroni-corrected for seven comparisons.

## Results

### Sensor Space

#### All Participants

**FIGURE 2:**
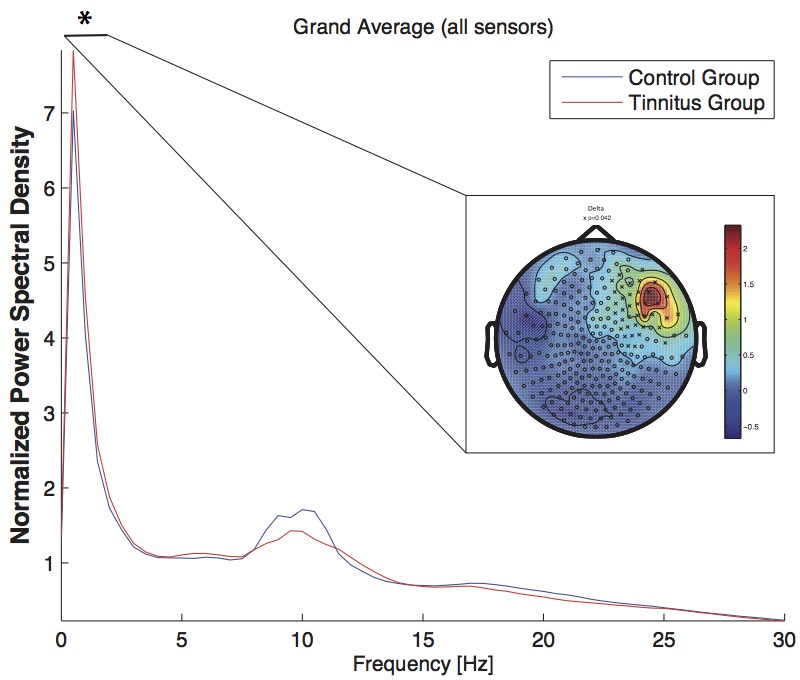
Power spectral density across all sensors for the tinnitus group (blue line) and the control group (red line). The power spectral density was averaged across all epochs and was normalized by the overall spectral power (channels × frequency bins). The significant difference (^∗^) in the delta band is depicted as topographic plot (tinnitus minus controls). The circles (‘o’) mark a sensor, the cross (‘x’) stands for a sensor, where a significant difference was found between the groups. The spectrum was cut off at 30 Hz to highlight the relevant frequency bands.

Visual inspection of the spectral power resembles the power distribution reported by Weisz et al. (2005), indicating an overall reduced alpha power in the tinnitus group and a slight increase at delta frequencies (cf. **Figure 2**) compared to the control group. A significant difference in the delta frequency band (*p* = 0.048) was located at a right fronto-temporal area (cf. **Figure 2**). In spite of its visual appearance, groups did not differ in the alpha band (8–12 Hz, *p* = 0.18) nor in the alpha sub-bands (low alpha; 8–10 Hz, *p* = 0.178, high alpha; 10–12 Hz, *p* = 0.247).

Groups did also not differ in the other frequency band (theta, beta, gamma; all *p* > 0.05). To explain the discrepancy between the visualized data (the plot, see **Figure 2**) and the statistics, we did a *post hoc* analysis of the alpha power, which displayed a considerable variance of the alpha power values in the patient group and therefore impeding the statistics to become significant.

#### Novices Only

**FIGURE 3:**
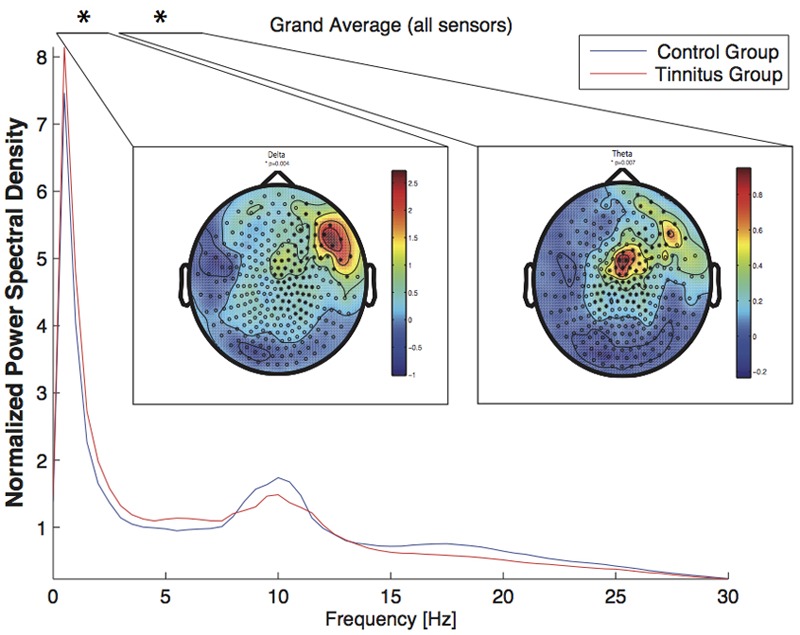
Power spectral density across all sensors for the *Novices* group (tinnitus subjects: blue line; control group: red line). The power spectral density was averaged across all epochs and was normalized by the overall spectral power (channels × frequency bins). The significant difference (^∗^) in the delta and the theta band is depicted as topographic plot (tinnitus minus controls). The circles (‘o’) mark a sensor, the star (‘^∗^’) stands for a sensor, where a significant difference was found between the groups. The spectrum was cut off at 30 Hz to highlight the relevant frequency bands.

Again, per visual inspection, the alpha frequency power in the novices (tinnitus vs. no tinnitus) appears to be different compared to those of the group of all subjects, yet this could not be affirmed through our statistical analysis. In the analysis of the MEG-Novices (tinnitus vs. no tinnitus) we found a significant difference in the delta (1–4 Hz, *p* < 0.01) and theta (4–8 Hz, *p* < 0.01, see **Figure 3**) frequency band. Again, all other frequency bands did not differ significantly between tinnitus subjects and healthy controls (*p* > 0.05).

#### Psychological Comorbidity Free Subjects Only

**FIGURE 4:**
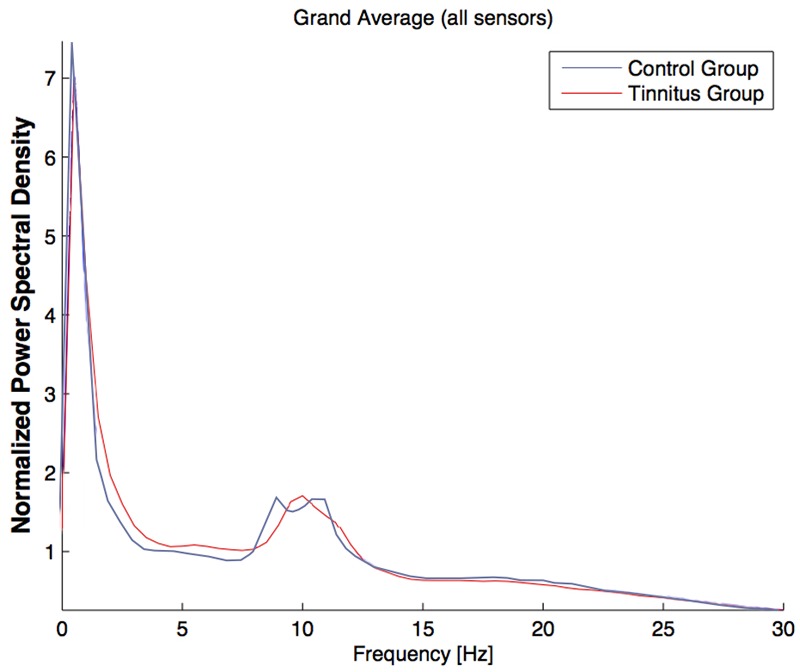
Power spectral density across all sensors for the *Psychologically Healthy* group (tinnitus subjects: blue line; control group: red line). The power spectral density was averaged across all epochs and was normalized by the overall spectral power (channels × frequency bins). The spectrum was cut off at 30 Hz to highlight the relevant frequency bands.

No significant differences between the group of mentally healthy tinnitus subjects and subjects without tinnitus occurred in the oscillatory resting state activity using the permutation testing on sensor level (*p* > 0.05, see **Figure 4**).

### Source Space

#### All Participants

**FIGURE 5:**
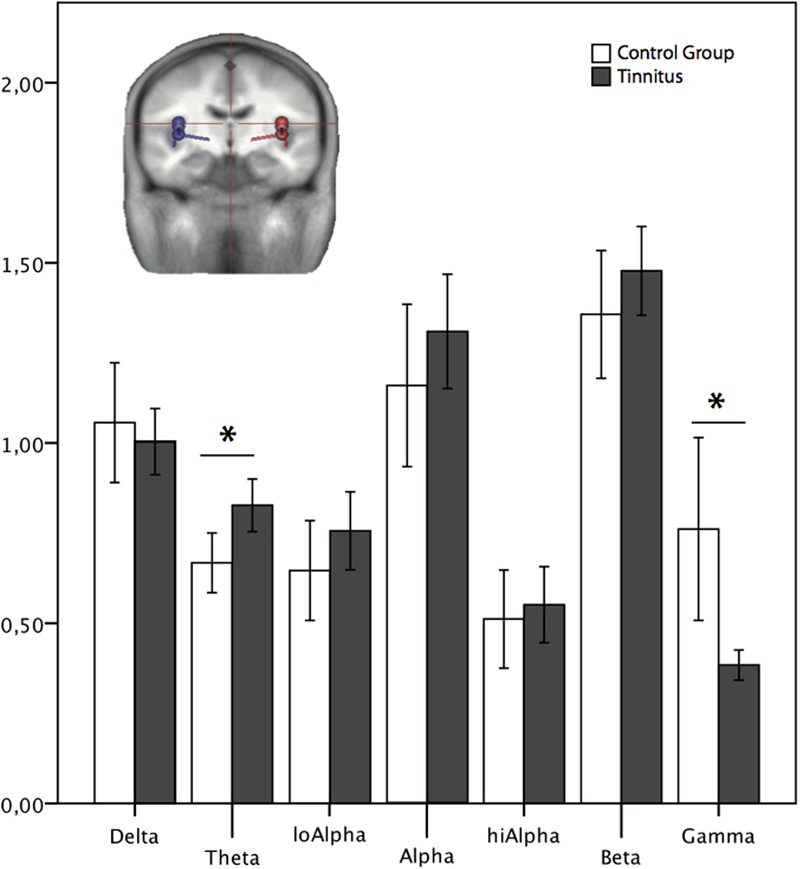
Normalized power spectrum of the source waveforms, reconstructed in auditory areas. ^∗^Marks a significant difference between the groups. The dipoles (blue: left, red: right side) in the head scheme show exemplarily four of six locations of the source montage. The same locations and orientations were used for both groups. LoAlpha = low alpha frequency range (8–10 Hz); hiAlpha = High alpha frequency range (10–12 Hz). Error bars symbolize ±2 standard errors.

When comparing the complete tinnitus group against the healthy controls using the BESA source reconstruction of oscillatory activity in the auditory areas, we find a significant difference in the theta and gamma band [one-way ANOVA with group as factor; theta: *F*(1,86) = 8.403, *p* = 0.005; gamma: *F*(1,86) = 9.408, *p* = 0.003, see **Figure 5**]. No significant difference emerged in the other frequency bands [*F*(1,86) < 1.603, *p* > 0.209].

#### Novices Only

**FIGURE 6:**
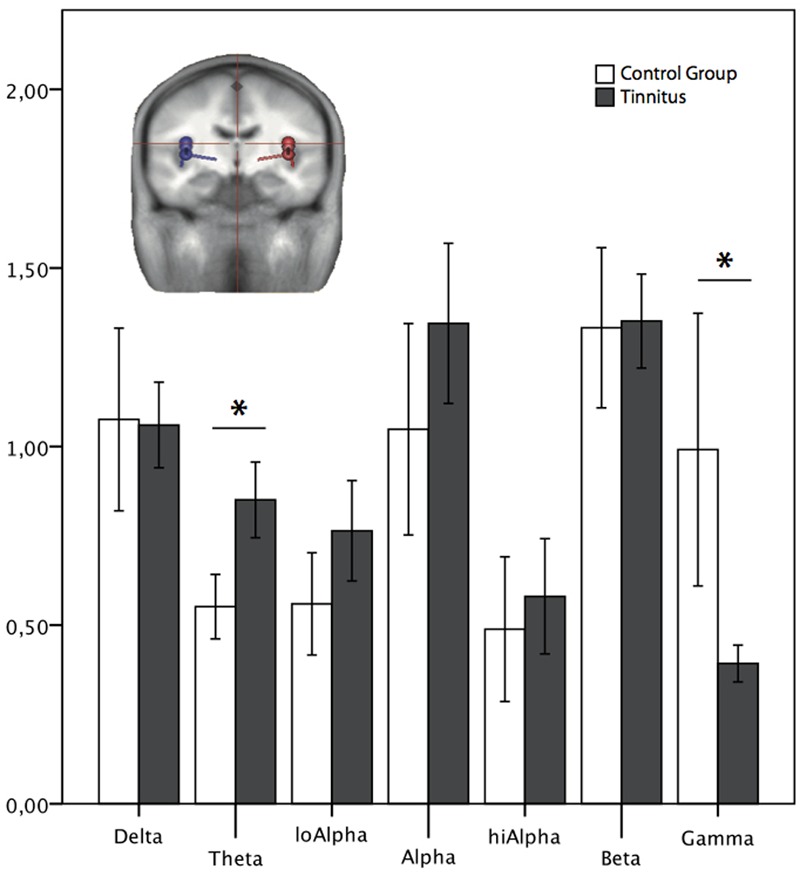
Normalized power spectrum of the source waveforms, reconstructed in auditory areas for the group of *Novices* only. ^∗^ Marks a significant difference between the groups. The dipoles (blue: left, red: right side) in the head scheme show exemplarily four of six locations of the source montage. The same locations and orientations were used for both groups. LoAlpha = low alpha frequency range (8–10 Hz); hiAlpha = high alpha frequency range (10–12 Hz). Error bars symbolize ±2 standard errors.

A significant difference resulted in the theta and gamma band in the temporally located sources [theta: *F*(1,53) = 18.026, *p* < 0.001; gamma: *F*(1,53) = 10.760, *p* = 0.002; see **Figure 6**]. The other frequency bands did not differ significantly (*p* > 0.05).

#### Psychological Comorbidity Free Subjects Only

**FIGURE 7:**
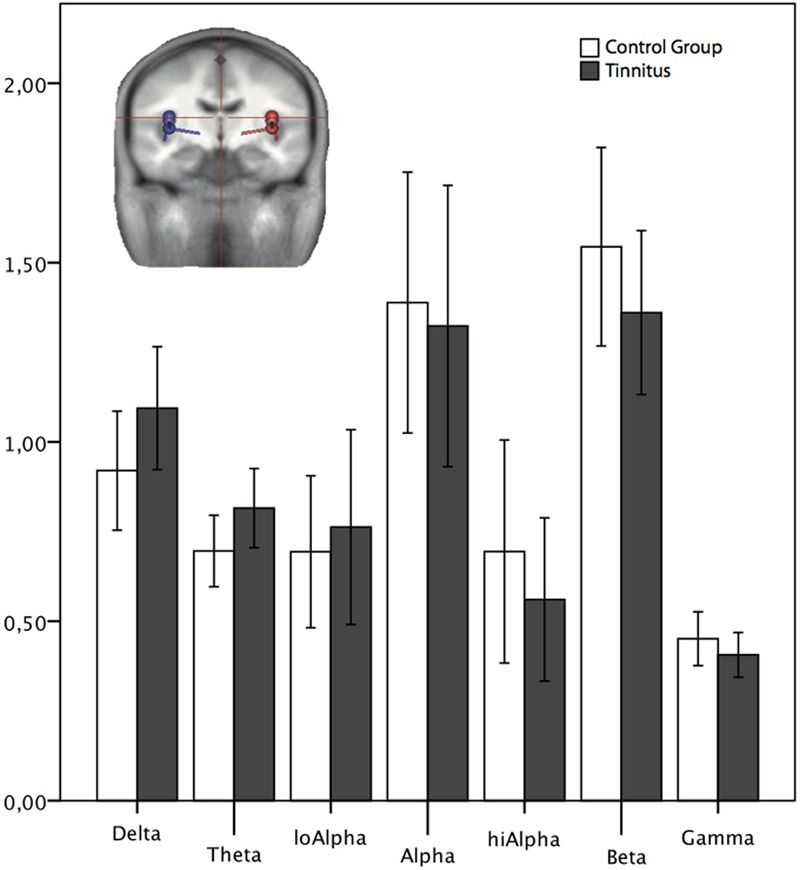
Normalized power spectrum of the source waveforms, reconstructed in auditory areas for the group of *psychologically healthy*. The dipoles (blue: left, red: right side) in the head scheme show exemplarily four of six locations of the source montage. The same locations and orientations were used for both groups. LoAlpha, low alpha frequency range (8–10 Hz); hiAlpha, high alpha frequency range (10–12 Hz). Error bars symbolize ±2 standard errors.

There was no significant difference for any of the frequency bands between the control and the experimental group for the group, in which psychological disorders were measured and excluded [*F*(1,26) < 2.587; *p* > 0.05, see **Figure 7**].

## Discussion

To the best of our knowledge, this is the first study investigating the neural oscillations of a sample comprising 88 subjects with and without tinnitus. Due to this large sample size, results of the current study can be assumed to be valuable with regard to questions concerning the role of neural oscillations in tinnitus.

Encompassing the whole group of participants, we found an increase in the delta spectrum in sensor space in the right frontal–temporal area of the coils. This means we found differences in the slow wave power of delta between the tinnitus and the control group in the directly picked up signal, the sensor space. The depicted activity here could correspond to auditory areas, but other sources are also imaginable. When localizing the origin of the signal within the brain, the analysis in source space, we found a difference when focusing on the auditory cortical areas for theta, another slow wave frequency. Sensor and source space findings agree in the coinciding occurrence of low oscillatory frequencies. Besides this, we found a difference in high frequency activity, namely a significant reduction in gamma band in source space, which is not detectable in our sensor space analysis.

Following the goal to rule out one confounding factor by restricting the analysis to subjects from both groups who were in MEG for the first time, the novices, we saw similar results: again, we found the difference in delta activity in sensor space, but additionally also in the theta frequency band. Furthermore, in source space we found a significant difference in the theta and gamma band in areas associated with auditory processing.

When analyzing the subgroup of subjects, who had been screened for psychological disorders and had been adjudged to be without mental health comorbidity, we did not find any difference between the groups of tinnitus subjects and healthy controls neither in sensor nor in source space.

According to Zobay et al. (2015), the TCD model would rather predict differences in the theta than the delta band. Yet, delta and theta frequencies are thought to be originating from the same source and are often treated equally (Pierzycki et al., 2016). The increase in slow wave activity (delta/theta) is in line with the TCD as well as the predictive coding model and previous findings (Weisz et al., 2005; Adjamian, 2014; De Ridder et al., 2015; Sedley et al., 2016). And, according to these models, we would also have expected changes in the gamma activity. With respect to the model of De Ridder et al. (2015) one would expect an increase in gamma power, whereas the integrative model of Sedley et al. (2016) would be predict a gamma decrease in the tinnitus population. In our sample we found a reduction in gamma band activity in the tinnitus group. So, these findings would be in line with the theoretical assumptions of Sedley et al. (2016) that the mismatch in tinnitus patients is reduced. As described above, here prediction (‘tinnitus’) and the precursor would be consistent and therefore no mismatch would be elicited. Evidence for this assumptions is derived from intracranial measurements in a single-case study (Sedley et al., 2015) as well as a group study (Sedley et al., 2012), where residual inhibition was used to manipulate the tinnitus. Additionally, a decrease in gamma activity was reported by Müller et al. (2013) after they used rTMS stimulation and was associated with an increase in tinnitus loudness. But gamma activity in general might be difficult to measure: research from other fields of domains, e.g., in the visual field (Muthukumaraswamy et al., 2010), has shown, how unpredictable and therefore problematic it is to trace gamma oscillations. Furthermore, gamma networks seem to be less reliable, too (Jin et al., 2011), which adds up to the intricacy of analyzing gamma activity. This might also explain, why we found the gamma results in source space only. In source space we have a better signal to noise ratio and therefore were more prone to pick up subtle signals as presumably the gamma band.

*A priori* we defined our source of interest in the auditory areas and therefore limited out analysis to the estimation of this region. An analysis with less beforehand defined restrictions or even network analysis, could possibly add insight to the findings of this paper. Especially, a replication of the findings of the gamma activity would be needed to validate our results. As shown in this paper, calculating both analysis (sensor and source space) is fruitful, as they show overlapping, yet not redundant results, which can trigger more comprehension.

To shed light on possible confounders which influence the neuronal activity we examined the factor of “being a novice.” Next to a different result in sensor space (finding a difference in theta and delta band), we saw in source space the same results as for the whole group. A comparison between novices and non-novices within the two groups (looking at tinnitus novices vs. tinnitus non-novices and the same for the control group) also would have been interesting; unfortunately these analyses were not possible as the two groups differed significantly in their age. In our sample, the novices were older than the non-novices. Students are our main resource of subjects and the novices were specifically recruited in the public and covered a broader age range. Concluding, our findings hint that this factor (“being a novice”) does not seem to have a meaningful impact on the results.

If we follow the above mentioned idea, that the more you control for confounders, the more precise are the results, this effect should also have been visible in the second subgroup, the group of mentally healthy participants. Conversely, this did not appear. As effects are expected to be subtle (Adjamian, 2014), the most probable reason for that might be the sample size of this subgroup (14 per group). This might have been too small to see a significant effect. Another possibility is, that, if you carefully control for mental health comorbidities, some oscillatory effects between the groups (tinnitus and non-tinnitus) disappear. Psychological disorders do reflect in the neuronal firing pattern and might account for some variance between the groups if comparing a tinnitus group against a group of non-tinnitus sufferers. The connection between tinnitus and psychological disorders is complex and its direction (e.g., causality, mediation, vulnerability) unclear. It is likely that comorbid psychological disorders might interfere with tinnitus perception, as postulated, e.g., for somatoform disorders (Signal-Filter-Modell according to Rief and Barsky) (Voderholzer and Hohagen, 2017). Here, the assumption is, that attentional processes, anxieties and depressive mood influence the cortical perception of the bodily symptoms. Next to this enhancement of bodily symptoms (the tinnitus correlate would most likely be tinnitus loudness), Zirke et al. (2010) state, that psychological comorbid disorders might hinder the habituation to the tinnitus itself. Furthermore, it is likely that the mental health status might also influence the overall distress perceived through the tinnitus. Handing out a questionnaire covering, e.g., the depressive symptomatic, is a good tool to estimate symptoms, yet these do not cover the full spectrum of mental health and are more a clue, not a tool to diagnose a person. Therefore the approach of this paper, performing the structured interviews covering the majority of psychological disorders, like the SKID or the CIDI (see Zirke et al., 2013), could be a step into the future to surely be aware of all mental health disorders in the participant group. The question, which oscillatory pattern between the two groups remains, if we control for as many confounders as possible, is yet to be answered.

Next to the two factors analyzed in this paper, many others may contribute to the heterogeneity within the measurement of neural oscillations. One step evolving from this issue is the development and handing out of questionnaires covering, e.g., cognitive states. Diaz et al. (2013) define the resting state “as a multi-faceted cognitive construct” and developed a questionnaire to quantify these cognitions. Seven dimensions were identified which were present during resting state measurements: discontinuity of mind, theory of mind, self, planning, sleepiness, comfort, and somatic awareness. Quantifying patient-control differences in the cognitive states might be a helpful tool for future measurements, to control more possible confounders and conduct measurements, which narrow down the true underlying difference of symptoms of tinnitus. To address this issue, other fields started projects of gathering multi-center data and meta data sharing in order to increase replicability and validity of the data (see Kafkafi et al., 2016).

One overall attempt to deal with the heterogeneity in tinnitus findings, is to define subtypes of tinnitus. TINNET (a European project on Tinnitus http://tinnet.tinnitusresearch.net) is aiming at quantifying and analyzing all aspects and facets of tinnitus and hopes to break down the heterogeneity. A workgroup of this project suggested guidelines for M/EEG measurements in tinnitus^1^. Those should make sure a better comparability of results between different labs and studies. The guideline advocates, next to other factors, the measurement of tinnitus distress, hearing loss, hyperacusis, and a resting state questionnaire. Taking them into consideration as confounders would have been helpful to rule out possible explanations. We think having quantified those factors would have helped to come to a more comprehensive and target-oriented interpretation of our results.

Another option to control for the heterogeneous characteristics in tinnitus research is to use a between-subjects-design with using tinnitus subjects only. Control group and tinnitus group may differ substantially in factors as, e.g., hearing loss or hyperacusis. With comparing subgroups within the group of tinnitus could therefore yield in more homogenous and more comparable groups. In this study we chose a comparison of tinnitus-experiencing subjects to subjects without tinnitus because we wanted to keep the comparability with other studies in the field (Weisz et al., 2005; Adjamian et al., 2012) and controlled that key aspects, as age and gender, are distributed equally in both groups.

Taken together, this study ruled out criticism of a too small sample size in the neuronal oscillations research in tinnitus. We were able to show that an analysis based on a sufficient sample size supports the significance of slow oscillatory frequencies and gamma band activity in the neuroscientific conceptualization of tinnitus. Yet, our data does not support a single model only. Targeting the heterogeneity of tinnitus on different levels, e.g., as focusing subtyping of tinnitus or, as done here, controlling more confounding variables, seems to be a necessary step to understand this phenomenon. To control the naiveté in studies is an approach to narrow down a manifold set of confounders (here: vigilance, arousal, stimulation due unfamiliarity to the situation). However, in our analysis of novices only, results did not hint toward a reduced variance in the sample and therefore to clearer results. Next to controlling factors forcefully (with excluding anyone who had experience in experiments beforehand or with a psychological comorbidity), quantifying the experience during the experiment is another option. This could, most likely, be done with questionnaires to assess those variable and take them into statistical analysis or at least into consideration when interpreting the findings. Controlling mental health comorbidities is, derived from the literature, clearly a worthy, yet very resource consuming approach. The lack of a statistical difference between the second subgroup in our study (*psychologically healthy*), could either show that whether or not those variables are controlled in a tinnitus sample, differences do disappear or that our sample size, thinned out through a relatively high drop-out rate and the time and a certain professional training demanding screening procedure, was too small to produce meaningful differences.

To sum up, we agree with previously published papers that neuronal oscillations in tinnitus are presently no distinctly defined biomarker (Pierzycki et al., 2016) and future research is needed to test the theoretically postulated models for tinnitus (e.g., TCD, predictive coding). Our study could contribute with highlighting the need of a thorough assessment of mental health in tinnitus studies and the rather minor importance of the factor ‘being a novice’ to the measurement. Overall, we could demonstrate a support for low neuronal frequencies as well as high frequencies to be connected with the percept of tinnitus.

## Author Contributions

PL, RW, AE, AL, and MJ recruited the participants. PL and AW developed and performed the data analysis. PL, AW, AE, RW, AL, and CP wrote the manuscript. All authors approved the final version of the manuscript.

## Conflict of Interest Statement

The authors declare that the research was conducted in the absence of any commercial or financial relationships that could be construed as a potential conflict of interest.

## Footnotes

1 http://tinnet.tinnitusresearch.net/images/pdf/WG3/Standardisation_Report_V5.pdf

## Supplementary Material

FIGURE S1 Location of the AEP montage by BESA covering the auditory areas.
