# Supplementary figures and images for: Targeting Heterogeneous Findings in Neuronal Oscillations in Tinnitus: Analyzing MEG Novices and Mental Health Comorbidities
Source: Front Psychol. 2018 Mar 2;9:235. doi: 10.3389/fpsyg.2018.00235 (PMC5841018; doi:10.3389/fpsyg.2018.00235)

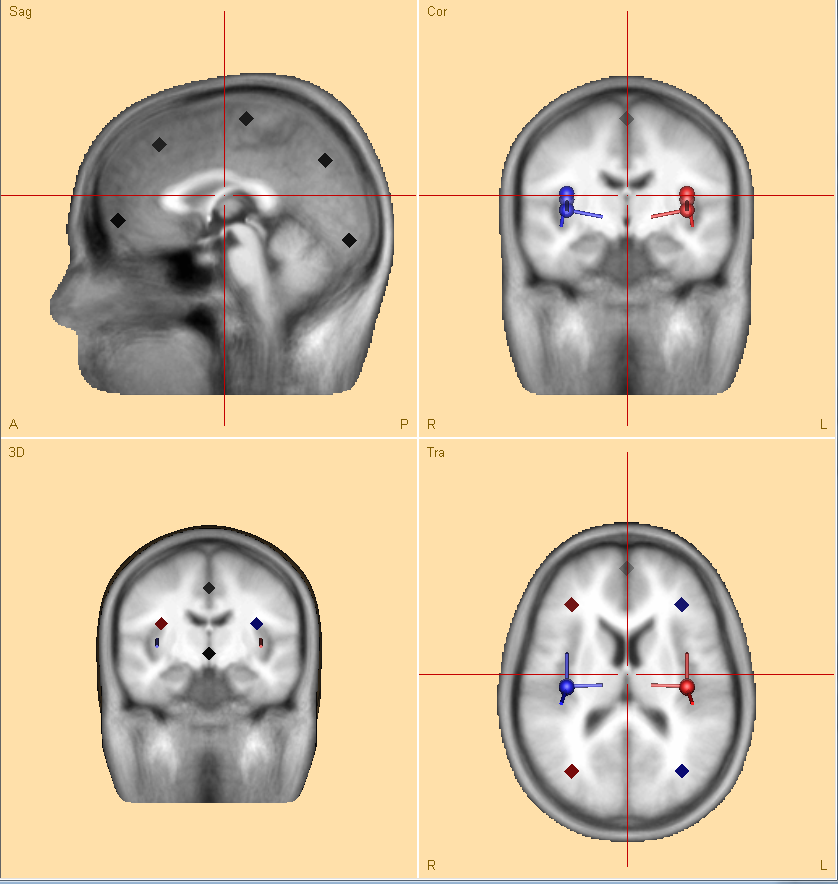

Supplement: FIGURE S1 — Location of the AEP montage by BESA covering the auditory areas. [file Image_1.PNG]
